# Genetic diversity of a Korean echovirus 5 isolate and response of the strain to five antiviral drugs

**DOI:** 10.1186/1743-422X-8-79

**Published:** 2011-02-24

**Authors:** Kwisung Park, Jaehyoung Song, Kyoungah Baek, Changgyun Lee, Donguk Kim, SamHyun Cho, JoonSoo Park, YoungJin Choi, Byunghak Kang, Hwajung Choi, Doo-Sung Cheon

**Affiliations:** 1 Department of Microbiology, Chungcheongnam-Do Institute of Health and Environmental Research, Daejeon, Korea; 2 Department of Herbal Resources, Professional Graduate School of Oriental Medicine, Wonkwang University, Iksan, Korea; 3 Department of Obstetrics and Gynecology, College of Medicine, Hanyang University, Seoul, Korea; 4 Department of Pediatrics, College of Medicine, Soonchunhyang University, Cheonan, Korea; 5 Department of Laboratory Medicine, College of Medicine, Soonchunhyang University, Cheonan, Korea; 6 Department of Enteric and Hepatitis Viruses, National Institute of Health, Korea Center for Disease Control and Prevention, Seoul, Korea; 7 Department of Clinical Pathology, Daejeon Health Science College, Daejeon, Korea

## Abstract

An outbreak of echovirus 5 (ECV 5) occurred in Korea in 2006, marking the first time this virus had been identified in the country since enterovirus surveillance began in 1993. Using a sample isolated from a young male patient with aseptic meningitis, we performed sequencing of the Korean ECV 5 strain and compared it with a prototype strain (Noyce). At the nucleotide level, the P1 region (85.3%) had the highest identity value; at the amino acid level, the P3 region (98.0%) had the highest identity value. The two strains shared all cleavage sites, with the exception of the VP1/2A site, which was TY/GA in the Noyce strain but TR/GA in the Korean ECV 5 isolate. In Vero cells infected with the Korean ECV 5 isolate, no cytotoxicity was observed in the presence of azidothymidine, acyclovir, amantadine, lamivudine, or ribavirin, when the drugs were administered at a CC_50 _value >100 μg/mL. Of the five drugs, only amantadine (IC_50_: 1 ± 0.42 μg/mL, TI: 100) and ribavirin (IC_50_: 22 ± 1.36 μg/mL, TI: 4.55) had any antiviral activity against the Korean ECV 5 isolate.

## 1. Introduction

Human enteroviruses (HEV) are RNA viruses from the Picornaviridae family. The 80 immunologically-distinct serotypes that are known to cause infections in humans can be grouped as follows: polioviruses (PV), echoviruses (ECV), coxsackieviruses A (CVA), coxsackieviruses B (CVB), and enterovirus (EV) types 68-71. These viruses are also classified genetically into five species (HEV-A to HEV-D and PVs). HEV-B group containing ECV 5 are CBV 1 to 6, CVA9, ECV 1 to ECV 7, ECV 9, ECV 11 to ECV 21, ECV 24 to ECV 27, ECV 29 to 33, EV 69, EV 73 [1-5].

ECV cause the same types of infections in humans as the CVB group, but are given a distinct classification primarily because they lack pathogenicity in newborn mice [6]. There are, however, strains of ECV that are pathogenic in mice [7]. ECV 5 infections have been associated with a wide variety of neurological and exanthematic diseases. The prototype strain of ECV 5 was isolated from a patient with aseptic meningitis and was later grouped as the fifth enterovirus serotype [8]. An outbreak of aseptic meningitis caused by ECV 5 occurred in Korea in 2006, marking the first time that ECV 5 had been identified in the country since enterovirus surveillance began in 1993 [9].

The ECV 5 genome contains approximately 7,500 nucleotide-long single-stranded RNA molecules with polarity and carries a small viral peptide (VPg) covalently attached to its 5' end. The 5' untranslated region (UTR) of the RNA is approximately 700 nt in length and is unusually long compared with the homologous region of cellular mRNA. The internal ribosomal entry site (IRES) was discovered in the 5' UTR of the HEVs. In these viruses, the IRES can fold to be a functional secondary RNA structure and drive translation initiation [10]. The coding region encompasses a single open reading frame that encodes a polyprotein divided into three sub-regions, P1, P2, and P3. The P1 region encodes the genetic information of four structural proteins, VP4, VP2, VP3, and VP1. The non-structural proteins are encoded in the P2 (2A-2C) and P3 (3A-3D) regions. A short 3' UTR of approximately 100 nt separates the coding region from the poly (A) tail [6,11].

At present, some 40 antiviral drugs have been formally licensed for use in humans, mostly for treatment of human immunodeficiency virus, hepatitis B virus, and herpesvirus infections. The number of antiviral drugs that have been licensed for use in treating highly-pathogenic RNA virus infections is very limited; the current list of approved drugs includes anti-influenza medications, M2 channel inhibitors (amantadine and rimantadine), and neuramidase inhibitors (oseltamivir and zanaminir). Ribavirin is licensed for the treatment of respiratory syncytial virus and hepatitis C virus infections [12,13]. Pleconaril, an antiviral drug developed in 1996 for treatment of diseases associated with picornavirus infections, can be used in treatments against enterovirus and rhinovirus infections [14]. However, the treatment of this drug was extremely limited and reported to pleconaril-resistant viruses [15,16].

In this study, the molecular biological characteristics and genetic diversity of Korean ECV 5 that do not exist the using antiviral agents for it but widespread currently were analyzed through the complete nucleotide sequencing and compared with ECV 5 prototype strain (Noyce). We also selected 5 kinds of antiviral drugs (azidothymidine, acyclovir, amantadine, lamivudine, and ribavirin) that were used as inhibitors of other viruses and examined for anti-viral activity for Korean ECV 5.

## 2. Materials and methods

### 2.1 Virus isolation and identification

The Korean ECV 5 strain was isolated from cerebrospinal fluid sampled from a male patient with aseptic meningitis who had been admitted to the Department of Pediatrics at the Soonchunhyang University Cheonan Hospital, Korea, in June 2006. The sample was inoculated into Vero cells and then incubated at 37°C with 5% CO_2 _until the appearance of cytopathic effects (CPE) took place. The identification of the Korean isolate was verified by a Basic Local Alignment Search Tool (BLAST) search in VP1 sequences; that is, the VP1 sequences of Korean isolate had the highest nucleotide similarity with ECV5 serotype strains [3].

### 2.2 Nucleotide sequencing and sequence analysis

The complete nucleotide sequence of the Korean ECV 5 strain was determined using a primer walking strategy; the sequences of the genome termini were determined by random amplification of cDNA ends (RACE) system (Invitrogen, Carlsbad, CA, USA). The PCR products were purified using a QIAquick PCR Purification Kit (Qiagen, Hamburg, Germany). The purified DNA was added to a reaction mixture containing 2 μL of Big Dye terminator reaction mix (ABI Prism BigDye Terminator Cycle Sequencing Kit; Applied Biosystems, Foster, CA, USA) and 2 pmoles of each primer. Sequencing reactions were subjected to an initial denaturation at 96°C for 1 min and 25 cycles consisting of 96°C for 10 sec, 50°C for 5 sec, and 60°C for 4 min in a Gene Amp PCR system 2700 (Applied Biosystems). The products were purified by precipitation with 100% cold ethanol and 3 M sodium-acetate (pH 5.8), then loaded on an automated 3100 Genetic Analyzer (Applied Biosystems).

Nucleotide sequences of enterovirus isolates were constructed to contig and were compared with a reference, the Noyce (Accession no. AF083069) strain, which was originally identified in the USA in 1954, and the nucleotide sequence of which was obtained from Genbank databases [17]. Sequence analyses were performed using computer software included in the Lasergene package (DNASTAR, Inc., Madison, WI, USA).

### 2.3 Antiviral drugs and antiviral activity assays

Assays of antiviral activity and cytotoxicity were evaluated by the modified sulforhodamine B (SRB) assay previously described and recently reported by Choi *et al *[18-20]. Ribavirin was purchased from DUCHEFA (Netherlands); azidothymidine, acyclovir, amantadine, and lamivudine were purchased from Sigma-Aldrich (St. Louis, MO, USA), as was SRB.

One day before infection, Vero cells were seeded onto a 96-well culture plate at a concentration of 2 × 10^4 ^cells per well. Next day, the medium was removed and then washed with 1 × phosphate buffered saline (PBS). Infectivity of the virus stock was determined by the SRB method and was determined as infectivity of the virus by SRB ID_50 _(50% infective dose). Following this, 0.09 mL of diluted virus suspension of ECV containing CCID_50 _(50% cell culture infective dose) of the virus stock to produce an appropriate cytopathic effects within 2 days after infection and 0.01 mL of medium containing an appropriate concentration of the compounds were added. The antiviral activity of each test material was determined with a 10-fold diluted concentration ranging from 0.1 to 100 μL/mL. Three wells were used as virus controls (virus-infected non-drug-treated cells) while three wells were used as cell controls (non-infected non-drug-treated cells). The culture plates were incubated at 37°C in 5% CO_2 _for 2 days. After washing once with 1 × PBS, 100 μL of cold (-20°C), 70% acetone was added to each well and left for 30 min at -20°C. 70% acetone was removed and 96-well plates were left at dry oven for 30 min. 100 μL of 0.4% (w/v) SRB in 1% acetic acid solution was added to each well and left at room temperature for 30 min. Unbound SRB was removed and the plates were washed 5 times with 1% acetic acid before oven drying and were then left in a dry oven for 1 day. Bound SRB was solubilized with 100 μL of 10 mM unbuffered tris-base solution and plates were left on a table for 30 min. The absorbance was read at 540 nm by using a VERSAmax microplate reader (Molecular Devices, Palo Alto, CA, USA) with a reference absorbance at 620 nm. To calculate the IC_50 _(50% inhibitory concentration) values, the results were transformed to percentage of controls and the IC_50 _values were graphically obtained from the dose-response curves. The percent protection achieved by the test compound in ECV-infected cells was calculated by the following formula:

$$\frac{{\left({\text{OD}}_{\text{t}}\right)}_{\text{ECV 5}}-{\left({\text{OD}}_{\text{c}}\right)}_{\text{ECV 5}}}{{\left({\text{OD}}_{\text{c}}\right)}_{\text{mock}}-{\left({\text{OD}}_{\text{c}}\right)}_{\text{ECV 5}}}\times \text{1}00\text{ (Expressed in }\%\text{)}$$

where (OD_t_)_ECV _is the optical density measured with a given concentration of the test compound in ECV-infected cells; (OD_c_)_ECV _is the optical density measured for the control untreated ECV-infected cells; and (OD_c_)_mock _is the optical density measured for control untreated mock-infected cells. The therapeutic index was defined as 50% cytotoxic concentration (CC_50_)/IC_50_.

### 2.4 Cytotoxicity assay

Vero cells were seeded into a 96-well culture plate at a concentration of 2 × 10^4 ^cells per well. Next day, the medium was removed and the 96-well plates were replaced with media containing the serially diluted compounds, and the cells were further incubated for 48 hrs. The culture medium was removed and washed with 1 × PBS. The next step was conducted by antiviral activity assay described above. To calculate the CC_50 _values, the results were transformed to percentage of controls and the CC_50 _values were graphically obtained from the dose-response curves.

## 3. Results

### 3.1 Complete nucleotide sequence of Korean ECV5

The Korean ECV 5 genome was sequenced (GenBank accession number HM775882) and its amino acid sequence was deduced. The genome is 7,430 nt in length, excluding the poly(A) tail. The 5'NCR contains 738 nt, followed by an ORF that encodes a viral polyprotein consisting of 2,196 codons, between a start codon (AUG) at position 739 and a stop codon (UGA) at position 7,326. The 3'NCR is 104 nt in length (Table 1).

**Table 1:** Genome components of the Korean echovirus 5 (ECV 5) isolate and comparison of predicted N-terminal cleavage sites for the Korean ECV 5 isolate and the Noyce strain

Region	Nucleotide sequence			Amino acid sequence			Predicted N-terminal cleavage sites	
			
	start	end	length	start	end	length	Kor-ECV5	Noyce
5'NCR	1	738	738	-	-	-	-	-
Polyprotein	739	7326	6588	1	2196	2196	-	-
P1	739	3324	2586	1	862	862	-	-
P2	3325	5058	1734	863	1440	578	TR/GA	TY/GA
P3	5059	7326	2268	1441	2196	756	FQ/GP	FQ/GP
VP4	739	945	207	1	69	69	-	-
VP2	946	1731	786	70	331	262	LN/SP	LN/SP
VP3	1732	2448	717	332	570	239	PQ/GL	PQ/GL
VP1	2449	3324	876	571	862	292	LQ/GD	LQ/GD
2A	3325	3774	450	863	1012	150	TR/GA	TY/GA
2B	3775	4071	297	1013	1111	99	EQ/GV	EQ/GV
2C	4072	5058	987	1112	1440	329	RQ/NN	RQ/NN
3A	5059	5355	267	1441	1529	89	FQ/GP	FQ/GP
3B	5356	5421	66	1530	1551	22	FQ/GA	FQ/GA
3C	5422	5970	549	1552	1734	183	VQ/GP	VQ/GP
3D	5971	7326	1386	1735	2196	462	EQ/GE	EQ/GE
3'NCR	7327	7430	104	-	-	-	-	-

### 3.2 Genome comparison between Korean ECV5 and the Noyce strain

The Korean ECV 5 isolate genome was divided into five regions (5'NCR, P1, P2, P3, and 3'NCR) and aligned with the Noyce strain using Megalign (DNASTAR). The P1 region (85.3%) had the highest level of nucleotide identity, followed by the P3 region (84.8%), the 3'NCR (84.5%), the 5'NCR (81.8%), and the P2 region (80.0%). The P3 region (98.0%) had the highest level of amino acid identity, followed by the P1 region (97.7%), and the P2 region (96.9%).

Most of the cleavage sites were identical between Korean ECV 5 and the Noyce strain. The only exception was the cleavage site between VP1 and 2A, which was TY/GA in the Noyce strain, but TR/GA in the Korean ECV 5 isolate (Table 1).

### 3.3 Antiviral activity of Korea ECV5

**Figure 1:**
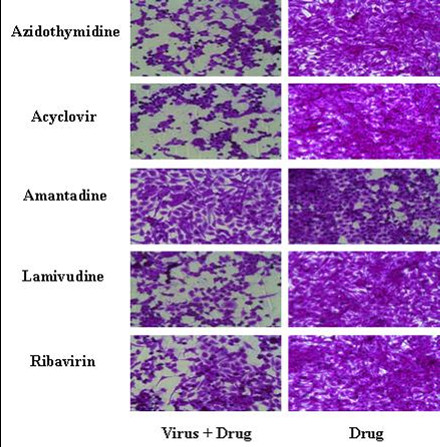
**Efficacy of antiviral agents at preventing cytopathic effects induced by infection with Korean echovirus 5**.

No signs of cytotoxicity were observed in Vero cells treated with any of the five antiviral drugs at a CC_50 _value >100 μg/mL. Amantadine and ribavirin exhibited antiviral activity against the Korean ECV 5 strain, though azidothymidine, acyclovir, and lamivudine did not. The amantadine possessed an IC_50 _value of 1 ± 0.42 μg/mL and a TI value of 100, while the ribavirin possessed an IC_50 _value of 22 ± 1.36 μg/mL and a TI value of 4.55 (Table 2 and Figure 1).

**Table 2:** Efficacy of antiviral drugs against Korean echovirus 5

Compounds	**IC**_**50**_^**a**^	**CC**_**50**_^**b**^	**TI**^**c**^
Azidothymidine	ND^d^	>100	-
Acyclovir	ND^d^	>100	-
Amantadine	1 ± 0.42	>100	100
Lamivudine	ND^d^	>100	-
Ribavirin	22 ± 1.36	>100	4.55

These results are the mean ± S.D. (n = 3)

^a ^Concentration of compound in μg/mL producing 50% inhibition of virus-induced cytopathic effects.

^b ^The 50% cytotoxic concentration for target cells in μg/mL.

^C ^Therapeutic index (TI) = CC_50_/IC_50_

^d ^Not Determined

## 4. Discussion

The ECV 5 is a rare enterovirus strain as causative aseptic meningitis and its sequence information is not common in the previous publication, including genbank database. This report describes the first complete nucleotide sequence for an ECV 5 isolated in Korea. Previous work has shown that sequence identity between different ECV serotypes is relatively high for the 5'NCR sequence, moderate for the P2 and P3 regions, and lowest for the P1 region [21]. This was not the pattern that emerged when we compared the Korean ECV 5 isolate and the Noyce strain; rather, the 5'NCR and P2 regions had relatively low nucleotide sequence identities (<81.8%), while the identities of the P1 and P3 regions were relatively high (>84.5%). Amino acid sequences for the protein coding regions had much higher sequence identities (>96.9%). Cleavage site variations have often been reported for VP2/VP3, VP3/VP1, and VP1/2A [21,22]. Thus, we were not surprised to find that, though conservation was observed at most sites, there had been a substitution (TY/GA→TR/GA) at VP1/2A. There was about 20% genetic difference between prototype and the current widespread strain, and its difference was found at the cleavage site. Therefore, development and screening of antiviral drugs have to be focused on the object of the current epidemic strain.

Of the five antiviral drugs tested here (azidothymidine, acyclovir, amantadine, lamivudine, and ribavirin), only amantadine (IC_50_: 1 μg/mL) and ribavirin (IC_50_: 22 μg/mL) had antiviral activity against Korean ECV 5, with amantadine showing stronger effects than ribavirin. Therefore, the amantadine and ribavirin could be applied to patients infected with ECV 5. It was reported that the amantadine could suppress the IRES mediated translation, and ribavirin is a nucleoside analogue with broad-spectrum antiviral activity by decreasing viral replication in EV71 [23,24].

In conclusion, this manuscript is the first report of the complete nucleotide sequence of the Korean ECV strain, as well as the first examination of its response to various antiviral drugs. This data should be useful in preventing future outbreaks of ECV5 and in treating patients infected with the strain. Accordingly, it is necessary to test more of the same kind of antiviral drugs and various enterovirus serotypes in future studies.

## 5. Competing interests

The authors declare that they have no competing interests.

## 6. Authors' contributions

KSP, JHS and KAB performed genome analysis and antiviral activity tests. CGL and DUK drafted the manuscripts. JSP, SHC and YJC contributed to collection specimen and clinical diagnosis. BHK, HJC and DSC designed the study and critically revised the manuscript. All of the authors read and approved the final version of the manuscript.

## References

[B1] Huttunen P Santti J Pulli T Hyypiä T The major echovirus group is genetically coherent and related to coxsackie B viruses J Gen Virol 1996 77 715 725 10.1099/0022-1317-77-4-715 8627260

[B2] Mayo M Pringle CR Virus taxonomy J Gen Virol 1998 79 649 657 9568957 10.1099/0022-1317-79-4-649

[B3] Oberste MS Maher K Kilpatrick DR Pallansch MA Molecular evolution of the human enteroviruses: Correlation of serotype with VP1 sequence and application to picornavirus classification J Virol 1999 73 1941 1948 9971773 10.1128/jvi.73.3.1941-1948.1999 PMC104435

[B4] King AMQ Brown F Christian P Hovi T Hyypiä T Knowles NJ Lemon SM Minor PD Palmenberg AC Skern T Picronaviridae Stanway G Van Regenmortel MHV, Fauquet CM, Bishop DHL, Carstens EB, Estes MK, Lemon SM, Maniloff J, Mayo MA, McGeoch DJ, Pringle CR, Wickner RB Virus taxonomy Seventh report of the International Committee on Taxonomy of Viruses 2000 Academic Press, San Diego, Calif 657 678

[B5] Stanway G Brown F Christian P Hovi T Hyypiä T King AMQ Knowles NJ Lemon SM Minor PD Pallansch MA Palmenberg AC Skern T Fauquet CM, Mayo MA, Maniloff J, Desselberger U, Ball LA Family Picornaviridae Virus Taxonomy Eighth Report of the International Committee on Taxonomy of Viruses 2005 Elsevier Academic Press, London 757 778

[B6] Melnick JL Fields BN, Knipe DM, Howley PM, Channock RM, Melnick JL, Monath TP, Roizman B, Straus SE Enteroviruses: polioviruses, coxsackieviruses, echoviruses, and newer enteroviruses Fields Virology 1996 3 Philadelphia: Lippincott-Raven Publishers 655 712

[B7] Eggers HJ Sabin AB Factors determining pathogenicity of variants of ECHO 9 virus for newborn mice J Exp Med 1959 110 951 967 10.1084/jem.110.6.951 13819536 PMC2137040

[B8] Melnick JL Tissue culture techniques and their application to original isolation, growth, and assay of poliomyelitis and orphan viruses Ann NY Acad Sci 1955 61 754 772 10.1111/j.1749-6632.1955.tb42532.x 13340582

[B9] Baek K Park K Jung E Chung E Park J Choi H Baek S Jee Y Cheon D Ahn G Molecular and epidemiological characterization of enteroviruses isolated in Chungnam, Korea from 2005 to 2006 J Microbiol Biotechnol 2009 19 1055 1064 10.4014/jmb.0810.584 19809266

[B10] Martínez-Salas E The impact of RNA structure on picornavirus IRES activity Trends Microbiol 2008 16 5 230 237 18420413 10.1016/j.tim.2008.01.013 PMC7172834

[B11] Hyypiä T Hovi T Knowles NJ Stanway G Classification of enteroviruses based on molecular and biological properties J Gen Virol 1997 78 1 11 9010279 10.1099/0022-1317-78-1-1

[B12] De Clercq E Antiviral drugs in current clinical use J Clin Virol 2004 30 115 133 10.1016/j.jcv.2004.02.009 15125867

[B13] Leyssen P De Clercq E Neyts J Molecular strategies to inhibit the replication of RNA viruses Antiviral Res 2008 78 9 25 10.1016/j.antiviral.2008.01.004 18313769 PMC7114363

[B14] Berg AK Olsson A Korsgren O Frisk G Antiviral treatment of Coxsackie B virus infection in human pancreatic islets Antiviral Res 2007 74 65 71 10.1016/j.antiviral.2006.12.001 17239967

[B15] Pevear DC Tull TM Seipel ME Groarke JM Activity of pleconaril against enterovirus Antimicrob Agents Chemother 1999 43 2109 2115 10471549 10.1128/aac.43.9.2109 PMC89431

[B16] Pevear DC Hayden FG Demenczuk TM Barone LR McKinlay MA Collett MS Relationship of pleconaril susceptibility and clinical outcomes in treatment of common colds caused by rhinoviruses Antimicrob Agents Chemother 2005 49 4492 4499 10.1128/AAC.49.11.4492-4499.2005 16251287 PMC1280128

[B17] Lindberg AM Johansson S Andersson A Echovirus 5: infectious transcripts and complete nucleotide sequence from uncloned cDNA Virus Res 1999 59 75 87 10.1016/S0168-1702(98)00127-0 10854167

[B18] Lin ZX Hoult JR Raman A Sulphorhodamine B assay for measuring proliferation of a pigmented melanocyte cell line and its application to the evaluation of crude drugs used in the treatment of vitiligo J Ethnopharmacol 1999 66 2 141 150 10.1016/S0378-8741(98)00199-8 10433470

[B19] Choi HJ Kim JH Lee CH Ahn YJ Song JH Baek SH Antiviral activity of quercetin 7-rhamnoside against porcine epidemic diarrhea virus Antiviral Res 2009 81 77 81 10.1016/j.antiviral.2008.10.002 18992773 PMC7114206

[B20] Choi HJ Song JH Park KS Kwon DH Inhibitory effects of quercetin 3-rhamnoside on influenza A virus replication Eur J Pharm Sci 2009 37 329 333 10.1016/j.ejps.2009.03.002 19491023

[B21] Lindberg AM Andersson P Savolainen C Mulders MN Hovi T Evolution of the genome of Human enterovirus B: incongruence between phylogenies of the VP1 and 3CD regions indicates frequent recombination within the species J Gen Virol 2003 84 1223 1235 10.1099/vir.0.18971-0 12692288

[B22] Andersson P Edman K Lindberg AM Molecular analysis of the echovirus 18 prototype: evidence of interserotypic recombination with echovirus 9 Virus Res 2002 85 71 83 10.1016/S0168-1702(02)00019-9 11955640

[B23] Chen YJ Zeng SJ Hsu JT Horng JT Yang HM Shih SR Chu YT Wu TY Amantadine as a regulator of internal ribosome entry site Acta Pharmacol Sin 2008 29 11 1327 1333 10.1111/j.1745-7254.2008.00876.x 18954527

[B24] Li ZH Li CM Ling P Shen FH Chen SH Liu CC Yu CK Chen SH Ribavirin reduces mortality in enterovirus 71-infected mice by decreasing viral replication J Infect Dis 2008 197 6 854 857 10.1086/527326 18279075 PMC7109938

